# Overview on the Antioxidants, Egg Yolk Alternatives, and Mesenchymal Stem Cells and Derivatives Used in Canine Sperm Cryopreservation

**DOI:** 10.3390/ani11071930

**Published:** 2021-06-28

**Authors:** Feriel Yasmine Mahiddine, Min-Jung Kim

**Affiliations:** Department of Research and Development, Mjbiogen Corp., Gwangnaru-ro 144, Seoul 14788, Korea; yasmini19@snu.ac.kr

**Keywords:** dog, semen, cryopreservation, assisted reproductive technology

## Abstract

**Simple Summary:**

Canine sperm cryopreservation is a method commonly used in veterinary clinics and laboratories. The present article reviews the antioxidants used in canine sperm cryopreservation, the egg yolk alternatives used for preventing cross-contamination, and the application of mesenchymal stem cells (MSCs) and their derivatives in dog sperm cryopreservation.

**Abstract:**

Sperm cryopreservation is a widely used assisted reproductive technology for canine species. The long-term storage of dog sperm is effective for the breeding of dogs living far apart, scheduling the time of artificial insemination that suits the female, and preventing diseases of the reproductive tract. However, spermatozoa functions are impaired during the freeze–thaw processes, which may decrease reproductive performance. Numerous attempts have been made to restore such impairments, including the use of cryoprotectants to prevent the damage caused by ice crystal formation, and supplementation of antioxidants to reduce reactive oxygen species generation due to osmotic stress during the procedure. Egg yolk derivatives, antioxidants, and, more recently, mesenchymal stem cells (MSCs) and their derivatives have been proposed in this research field. This review article will summarize the current literature available on the topic.

## 1. Introduction

The use of assisted reproductive technologies (ARTs) in canine species is becoming more frequent due to the possibility of reducing the omnipresent threat of canine reproductive disorders, such as brucellosis [1], venereal transmissible tumors [2]; overcoming the limitations related to the characteristics of the female reproductive cycles; shipping canine semen to other facilities; and enabling the breeding of dogs living far apart. Practitioners using ARTs in their clinics can schedule semen collection or artificial insemination (AI) according to the male’s general condition, the female’s estrus cycle or vaccination schedule, or a suitable parturition date. These options allow the clinicians and breeders to save both time and money, schedule a cesarean delivery if required, and avoid transmissible diseases. Dog sperm cryopreservation is a widely used ART in clinics. However, the post-thaw parameters of cryopreserved sperm are more variable and poorer than those of fresh sperm [3,4,5]. Therefore, a standard and an efficient cryopreservation protocol is warranted for canine species.

The damages caused by reactive oxygen species (ROS) and oxidative stress during the freezing process are the main obstacles to sperm cryopreservation [6,7]. Moreover, thermotropic phase transitions during cooling–thawing processes induce a reorganization of the lipid phase [8], with a subsequent loss of cholesterol and polyunsaturated fatty acids [9]. These modifications are also associated with an influx of intracellular calcium, which triggers protein phosphorylation and later sperm capacitation-like changes, also referred to as cryocapacitation [10,11]. To overcome this limitation, supplementation of various antioxidants in freezing extenders has been attempted for decades [12]. In addition, although egg yolk is the most widely used cryoprotectant for sperm, issues of cross-contamination and the manual separation of the egg yolk have been addressed [13,14]. To overcome these shortcomings, skim milk [15], Equex STM paste [16], low-density lipoproteins (LDLs) [17], and *Soybean lecithin* [13,18] have been proposed as alternatives to egg yolk in the cryopreservation buffer. Recently, mesenchymal stem cells (MSCs) and their derivatives have been used in sperm cryopreservation research [19]. In 1976, Friedenstein et al. isolated MSCs from bone marrow for the first time [20]. Since then, these cells have been used in clinical trials and proven useful for treating various conditions, such as arthritis, diabetes, cancer, and neurological disorders in humans [21]. Subsequently, the use of MSC derivatives in veterinary medicine has garnered much attention; however, their use in theriogenology remained limited compared to that in other clinical fields.

The present review discusses the current trends in cryoprotectants and the chemicals used in canine sperm cryopreservation, with a particular focus on antioxidant supplementation and egg yolk alternatives, and summarizes the innovative emerging technologies using MSCs and their derivatives.

## 2. Traditional Cryoprotectants Supplemented with Antioxidants

During cryopreservation, a sudden decrease in temperature induces deleterious changes in sperm function and morphology. This is due to the osmotic stress induced by ice crystal formation, which further promotes ROS production, membrane damage, and intracellular content leakage. To counteract these events, the supplementation of cryoprotectants and antioxidants is essential. In sperm cryopreservation, various chemicals have been tested for their antioxidant potential and proven useful in recovering optimal post-thaw sperm quality parameters in different species (Table 1). In canine sperm cryopreservation, effects of anti-oxidants such as butylated hydroxytoluene [22,23], taurine [24,25], vitamin E [24,26], and ascorbic acid [24,26,27] have been investigated and have similar effects on post-thaw sperm quality-related parameters. In this part, less known antioxidants will be reviewed, and their optimal concentrations and effects in sperm cryopreservation in dogs and other species will be compared (Table 1).

### 2.1. Astaxanthin

Astaxanthin is a red carotenoid used for its anti-cancer, antioxidant, anti-aging effects, and for its role in delaying or preventing degenerative conditions such as aging, eye diseases, or atherosclerosis [84,85]. In canine sperm cryopreservation, astaxanthin supplementation in the freezing media significantly enhanced the post-thaw quality-related sperm parameters [30]. Carotenoids are known for their antioxidant activity and are commonly taken as dietary supplements. However, the effects of dietary supplementation with carotenoids on sperm quality are variable and inconclusive in different species [86,87,88,89,90]. Astaxanthin exhibits the highest antioxidant activity among carotenoids [84], and its direct addition to the freezing media significantly protected the sperm parameters in dogs and other species as well (Table 1). Through these results, it can be concluded that carotenoids, especially astaxanthin, have a better protective effect on sperm parameters when added directly to the samples, than being included in the diet. Additionally, in boars, astaxanthin increased the sperm oocytes penetration ratio, in vitro fertilization (IVF) efficiency, and cleavage and blastocyst rates (Table 1). These results suggest that astaxanthin could be used to enhance the dog sperm fertilization rate, and more research using this chemical to enhance canine IVF is warranted.

### 2.2. Resveratrol

Resveratrol is a polyphenol found in plants, with powerful antioxidant effects. Dietary inclusion of resveratrol was associated with a higher oxidative stress resistance [91]. In canine sperm cryopreservation, supplementation of resveratrol in the freezing media improved the effects of cryopreservation [35]. Since in other species, resveratrol supplementation enhanced the antioxidant capacities (Table 1), resveratrol’s effects on canine sperm’s post-thaw parameters can be attributed to its antioxidant activity. Moreover, chromatin integrity was well preserved in the resveratrol-treated samples, which can enhance the male fertility potential [92]. However, its effect on oxidative stress and fertility in canine frozen–thawed sperm has not been assessed yet.

### 2.3. Quercetin

Quercetin is a flavonoid with anti-bacterial, anti-carcinogenic, anti-inflammatory, and antioxidant activities [93]. It has been used in canine sperm cryopreservation and protected the post-thaw quality-related kinematic parameters and fertility [39]. However, artificial insemination was used as a fertility assessment in this study, and an evaluation of in vitro fertilizing abilities is still needed to confirm quercetin’s effects on dog fertility [39]. Moreover, sperm viability, chromatin integrity, plasma membrane and acrosome integrity, oxidative stress levels, and mitochondrial activity were not assessed in this study. Therefore, more complete studies on its effect on canine sperm are needed.

### 2.4. Myoinositol

Myoinositol is an active form of inositol, which belongs to the vitamin B complex. It shows antioxidant properties and has been used to treat male infertility due to its enhancing effects on sperm quality [94,95]. Myoinositol is a component of the seminiferous tubule fluid, and it is involved in sperm maturation, capacitation, motility, mitochondrial function, viability, and acrosomal reaction [45,96]. It is also present in the seminal plasma, where it acts as an osmo-regulator [97]. In canine semen cryopreservation, the supplementation of myoinositol in the freezing medium significantly protected the post-thaw quality parameters from the effects of cryopreservation, including motility; linearity; straightness; amplitude of lateral head displacement; live sperm percentage; membrane, chromatin, and acrosome integrity; and mucus-penetrating ability in comparison with a control [42]. Therefore, myoinositol protects the sperm survival rate, hyperactivation, and kinematic parameters, making it a potent supplement for cryopreservation media due its enhancing effects on sperm fertilization ability. Additionally, at the gene level, myoinositol significantly decreased the expression of pro-apoptotic and mitochondrial ROS modulator genes and increased the expression of protamine and anti-apoptotic genes in sperm [42]. However, whether or not myoinositol had a real effect on sperm genes expressions is controversial, as mature sperm cells are presumed to have no translation machinery [98].

### 2.5. Curcumin

This polyphenol is the active ingredient in turmeric (*Curcuma longa*) and has been used for its therapeutical potential since ancient times [99]. Even though the protective and antioxidant effects of curcumin have been documented previously, its use in sperm cryopreservation is recent. In dogs, the effects of curcumin supplementation during sperm cryopreservation were evaluated recently [51]. In this study, the addition of curcumin significantly protected sperm the post-thaw kinematic parameters, DNA integrity, and oxidative defenses. Interestingly, the authors investigated NADPH oxidase 5 (NOX5) gene expression, which has been identified in human spermatocytes and spermatids [100] and has been linked to ROS production in human sperm but has not been studied in canine sperm yet. Curcumin supplementation resulted in low expression levels of NOX5 genes. However, as mentioned previously, cautions should be taken when discussing gene expression analysis results in spermatozoa.

### 2.6. Iodixanol

Traditionally, iodixanol is commonly used as a contrast medium [101]. Nowadays, it is also used in density-gradient centrifugation [102,103], and more recently for sperm cryopreservation [54,56]. In dogs, 1.5% iodixanol conserved post-thaw sperm motility and mucus-penetrating ability, relieved oxidative stress, and enhanced protamine-related gene expression in sperm [56]. Furthermore, it also reduced protamine deficiency levels, and the relative expression of pro-apoptotic and mitochondrial ROS modulator genes in sperm [56]. When incubated in a canine capacitation medium supplemented with iodixanol, the sperm viability and acrosome integrity were significantly higher than the control group [56]. The supplementation of a canine capacitation medium with iodixanol significantly improved the sperm viability and the acrosome integrity in comparison with the control. Overall, iodixanol protects the general post-thaw sperm kinematics and sperm during capacitation and can thus be used in canine sperm capacitation media.

### 2.7. Spermine

The seminal plasma is an important fraction of the semen, which is involved in sperm regulation, homeostasis, and ROS modulation [104]. Spermine is a polyamine present in the seminal plasma, which exerts protective effects on cells [105]. Despite its antioxidant properties, however, the sperm fraction is usually separated from the seminal plasma during cryopreservation. Consequently, spermine is withdrawn from the sperm [106]. This protein is specific to human and rat seminal plasma [107]. However, the addition of spermine to the canine sperm cryopreservation medium could regulate oxidative stress, conserve sperm motility while maintaining membrane integrity, and preventing apoptosis-like changes and the cryocapacitation of dog sperm [62]. In this study too, the gene expression of NOX5 and other genes associated with oxidative stress and apoptosis was lower when compared with the control group.

### 2.8. Kinetin

Kinetin, a member of the cytokinin family, exhibits immune and antioxidant potential [108]. However, studies reporting the use of kinetin as a supplement in ARTs are scarce. Kinetin is more commonly added to the embryo culture media for plants than for animals, and its use has been reported only once in porcine embryos [109]. In semen experiments, kinetin has been used once in canine sperm cryopreservation and once in ram sperm storage at a refrigerator temperature [65]. In dogs, kinetin could protect the sperm from oxidative stress and could significantly protect sperm kinematics, mitochondrial activity, and membrane and acrosome integrity [64]. Therefore, the use of kinetin as an antioxidant in sperm cryopreservation should be considered in future research.

### 2.9. Melatonin

This hormone is synthesized by the pineal gland and is involved in many biological and physiological processes [110]. Melatonin and its metabolites exert both direct and indirect antioxidant effects in cells. Melatonin is a potent antioxidant and free radical scavenger [110,111,112], and it is commonly used in ARTs [113,114,115] including sperm cryopreservation. The use of melatonin as an oral or in vitro supplement in semen experiments is well documented [116,117,118]. Its protective and beneficial effects on the sperm during cryopreservation have been confirmed in many species, including humans, stallions, bulls, rams, mice, pigs, rabbits [68,71,75,77,78,119,120], and, more recently, in dogs [69]. Melatonin alleviates oxidative stress and reduces ROS levels, plasma membrane lipid peroxidation, DNA fragmentation, and apoptosis-like changes [116]. In addition, it significantly protects post-thaw sperm motility, membrane and acrosome integrity, and mitochondrial activity [116]. In canine sperm cryopreservation, melatonin conserved the post-thaw sperm plasma membrane integrity, kinematic parameters, mitochondrial activity, and conception rates when compared to the control group [69]. However, more experiments with canine sperm are warranted to unveil the precise properties and actions of melatonin in this system.

### 2.10. Metformin

Despite being extracted from a toxic plant [121,122,123], metformin has been used to treat various disorders, including type 2 diabetes mellitus [124] and cancer [125]. In ART and reproductive biology, metformin has been used for its antioxidant and therapeutic properties [126]. However, it has only been used for sperm cryopreservation in mice [81] and canines [80]. In mouse sperm, metformin did not affect the post-thaw quality parameters, although a higher fertilization rate and lower abnormal zygote rate were recorded in the treatment groups [81]. In contrast, in dog sperm, the supplementation of cryopreservation medium with metformin protected the post-thaw quality markers and parameters, including motility and oxidative defense [80]. Subsequently, these beneficial effects of metformin on canine sperm parameters were proposed to be produced through the activation of the 5′ adenosine monophosphate-activated protein kinase (AMPK) pathway [80]. To date, that study remains the only one to demonstrate the beneficial effects of metformin on sperm kinematics. 

### 2.11. Olive-Derived Antioxidants

Among olive derivatives, 3,4-dihydroxyphenyl glycol (DHPG) is a phenol isolated from olive oil waste, with powerful antioxidant properties [127]. Its use in sperm cryopreservation is still new as it has only been used in two species (Table 1). In canine sperm cryopreservation, the addition of DHPG significantly protected the frozen–thawed sperm kinematic parameters, and reduced DNA damage [82]. The activities of enzymatic antioxidants were also enhanced in the treated samples. However, these results are not in accordance with the ones found in ram post-thawed sperm when the same concentrations were used (Table 1), and more studies are needed to confirm these results, and evaluate the effects of DHPG on in vivo and in vitro fertility. 

## 3. Egg Yolk Alternatives

The application of cryopreservation to store and preserve sperm cells requires the use of cryoprotectants. Glycerol, egg yolk, skim milk, and Equex (Sodium Dodecyl Sulfate-SDS) are the major cryoprotectants used in the current standardized protocols [128]. Typically, extenders used for cryopreservation can be prepared in laboratories or purchased from commercial suppliers. The extenders used for canine sperm require the addition of fresh egg yolk, as it is the most common external cryoprotectant used. However, the use of egg yolk is associated with potential cross-contamination, and the manual separation of egg yolk from albumen is difficult and time-consuming [129]. Therefore, an increasing number of recent studies have explored egg yolk alternatives and assessed their efficacy; however, effects of these alternatives on fertility rates have not yet been assessed.

### 3.1. Low-Density Lipoproteins

Egg yolk contains LDLs, which can prevent cholesterol efflux and lower tyrosine-containing protein phosphorylation, thereby inhibiting sperm capacitation [130]. The importance of LDL in sperm cryopreservation has been demonstrated, as it is the key component of egg yolk that is responsible for sperm protection [131,132]. In previous studies, 4–12% LDL in semen extenders for different species could achieve better results than the percentage of egg yolk typically used in extenders (20%) [17,130,133,134]. Several explanations and hypotheses regarding the mechanisms through which LDLs protect sperm have been proposed.

In canine sperm cryopreservation, 6% LDL in Tris–citric acid–fructose medium did not reduce the fertilization ability of spermatozoa [17]. Moreover, the use of 6% LDL instead of egg yolk successfully preserved the motility parameters and DNA, membrane, and acrosome integrity [17]. In another study, 6% LDL combined with 20 mmol/mL glutamine was more efficient in protecting sperm during cryopreservation than egg yolk, 6% LDL alone, or the Equex extender [16]. These results suggest that LDL addition to extenders is a promising alternative to the conventional use of egg yolk. However, LDL use is limited due to the complexity of its extraction and purification methods [135].

### 3.2. Egg Yolk Plasma

LDL represents the protective fraction of egg yolk, and a major portion of LDL is present in the plasma (approximately 85%) [129]. Liquefied or lyophilized egg yolk plasma can be used as a substitute for LDL in canine sperm cryopreservation. Unlike whole egg yolk, the plasma fraction can be sterilized using gamma irradiation. Egg yolk plasma has been proven efficient as a substitute for whole egg yolk in stallion sperm cryopreservation. Equine sperm cryopreservation medium kits that include egg yolk plasma are already being commercialized [129]. In canine sperm, egg yolk plasma was more efficient in preserving the post-thaw quality parameters than whole egg yolk [136,137]. Egg yolk plasma well preserved motility, morphology, and membrane and acrosome integrity when the sperm were cooled for up to 10 h before cryopreservation [136]. However, the precise mechanism and extended effects of egg yolk plasma on canine sperm cryopreservation remain unknown and warrant further research.

### 3.3. Soybean Lecithin

Lecithin contains 65% phospholipids and triglycerides and is a component of egg yolk and soybeans [138]. The lecithin extracted from soybean oil has traditionally been used in food and non-food products as a substitute for animal fat [138,139]. It can act as a thickener, emulsifier, or crystallization control agent [139]. Soybean lecithin has been proposed as an alternative to egg yolk and its derivatives in sperm freezing media, and many new extenders containing lecithin have been developed and used in different species, including rams, sheep, bovids, equines, boars, and humans [140,141,142,143,144,145,146]. Indeed, lecithin could shield sperm from physical damage by forming a protective layer, preventing ice crystal formation, and replacing sperm membrane phospholipids [147]. 

In dog sperm cryopreservation, high concentrations of lecithin (1% and 2%) showed no positive effects on the post-thaw quality parameters in comparison with the egg yolk extender [148,149]. When supplemented with 0.04% lecithin without Equex, the frozen–thawed canine sperm parameters were similar to the ones obtained with a Tris–egg yolk–Equex extender [150]. In other mammals, effective lecithin concentrations ranged between 0.08 and 6% [145,147,151,152]. At concentrations of 0.01, 0.05, or 0.1%, lecithin showed a similar protective ability to egg yolk for canine sperm [18]. Moreover, egg yolk exacerbated the lipid peroxidation, consequently increasing the stress susceptibility of the frozen samples [18,153]. Therefore, lecithin offers more advantages than the conventional whole egg yolk. In a previous study, however, the higher lecithin concentrations used (0.05–0.1%) were detrimental to the post-thaw quality parameters of sperm [18]. This confirms that low concentrations of lecithin should be used for canine sperm cryopreservation. 

### 3.4. Skim Milk

A skim milk extender is commonly used in mouse [154] and goat [155] sperm cryopreservation. It is free of lipids, and its protective constituent, casein micelles, reduces lipid loss and reduces the binding of seminal plasma proteins to sperm [132,156]. The main advantages of using skim milk-based extenders in comparison with egg yolk and its derivatives, are its simple storage conditions, cheap costs, availability, and easy acquisition. It has been reported as a good alternative to egg yolk in canine sperm cryopreservation and showed no adverse effect on in vivo fertility [15,157]. It was also hypothesized that the fertility of spermatozoa frozen with skim milk might be maintained longer than those frozen with egg yolk [15]. However, in vitro fertility assays should be performed to confirm these results.

### 3.5. Polyvinyl Alcohol

Polyvinyl alcohol (PVA) is a synthetic polymer with ice recrystallization inhibition properties in pure water, at concentrations as low as 1 mg/mL [158]. In an egg-yolk-free extender supplemented with PVA, the frozen–thawed canine sperm total motility, progressive motility, and acrosome integrity were protected [159]. However, the control group used in this study was an egg-yolk-free extender containing only glycerol as a cryoprotectant. More studies using egg-yolk-based extenders as a control group, and other conventional non-penetrant cryoprotectants, should be conducted. Moreover, sperm motility has been assessed by visual evaluation only, which does not give a full screening of the sperm kinematic parameters. Therefore, further evaluations of PVA effects on canine sperm cryopreservation are needed.

## 4. Cryoprotectants Supplemented with MSCs or Their Derivatives

MSCs exert their therapeutic effects through diverse mechanisms, which have been widely studied [160,161]. They can release factors involved in oxidative stress defense, apoptosis, cell survival, and cell metabolism [162]. They act through different pathways, such as the PI3K/Akt pathway that enhances MSCs survival ability [163], by activating ERK1/2 that promotes cell proliferation [164], or by suppressing the p38MAPK pathway to enhance oxidative defenses [164]. 

Since their discovery, MSCs have been extensively studied, characterized, and used in different clinical and experimental studies. The therapeutic potential of MSCs and their derivatives has been demonstrated in multiple studies, but their use in ARTs is still new and warrants further investigation to unveil their full potential (Table 2). Recently, an increasing number of studies have demonstrated the utility of MSCs as well as their conditioned media (CM) and extracellular vesicles in reproductive medicine and biotechnology, such as fertility recovery trials [165,166,167,168,169], sperm cryopreservation [19,170,171,172,173,174], oocytes in vitro maturation [175,176,177,178], and embryo production and maturation [167,177]. In canine sperm cryopreservation, the following two types of MSC have been used: adipose-derived MSCs (Ad-MSCs) [170,171] and amniotic-membrane-derived MSCs (AMSCs) [172,173].

### 4.1. Ad-MSCs Supplementation

In a recent study, Ad-MSCs were directly added to an egg yolk–Tris extender for the first time [171]; the results indicated that Ad-MSCs could preserve post-thaw viability; motility; and plasma membrane, chromatin, and acrosome integrity. Additionally, as a potential mechanism underlying this effect, the authors hypothesized that Ad-MSCs secrete repair factors in the medium to protect sperm cells from cryodamage [171], as the expression of genes related to membrane and chromatin repair was significantly enhanced when the sperm were treated with an extender supplemented with Ad-MSCs. These results confirm that Ad-MSCs can significantly enhance post-thaw sperm quality. However, the precise mechanisms involved remain unknown.

Of note is the fact that the main limiting factor of that study was the presence of cells other than sperm cells in the straws. Before analysis or use for insemination, the samples must be thoroughly washed; however, the risk of the contamination of sperm with Ad-MSCs remains high, and a new separation method is required. Furthermore, the packing proportion must be taken into account, as the sperm concentration in the straw is an important aspect of cryopreservation. The typical sperm concentrations used a range between 100 × 10^6^ and 200 × 10^6^ cells/mL, and this range is suitable for canine sperm cryopreservation protocols [181,182,183]. However, the addition of live cells to the medium alters the cell density, concentration, and nutrient availability. Indeed, in a previous study, the addition of high concentrations of Ad-MSCs (5 × 10^6^ cells/mL) increased the sperm cell damage and decreased the viability and kinematics [171]. A higher cell density promotes the production of ROS [184] and release of pro-apoptotic factors [185,186], producing negative effects on cell recovery [186,187].

### 4.2. Ad-MSCs Derivatives Supplementation

A good alternative to the use of stem cells-based therapies, is the use of their derivatives (conditioned medium or extracellular vesicles). Recently, several MSC derivatives have been extracted, characterized, and processed for use as alternatives to stem cells in various therapies [188]. The major advantages of these alternatives are that, compared with stem cells, their derivatives present greater innocuity, longer viability, and easier manipulation and storage, and also lack teratogenic potential [189,190,191,192]. 

Exosomes are single-membrane extracellular vesicles secreted by cells, with diameters ranging from 30 to 200 nm [193,194]. They transduce cellular messages to the neighboring cells and tissues and contain a broad array of proteins, RNAs, lipids, and DNA [194]. In sperm cryopreservation, the use of exosomes is advantageous because they do not cause an increase in cell density, which is detrimental, unlike the use of whole MSCs [171]. Exosomes derived from Ad-MSCs have been used in canine sperm cryopreservation and shown to successfully maintain high post-thaw quality [170]. The addition of 50 µg/mL exosomes to the freezing extenders positively affected sperm motility, progressive motility, acrosome and membrane integrity, and viability [170]. These protective effects were likely produced by the action of exosomal mRNAs and proteins. With the recent published data about extracellular vesicles’ uptake by spermatozoa [195,196], more research using them in sperm cryopreservation is expected. Nonetheless, further studies are warranted to evaluate the precise mechanism of action of exosomes, and their effects on fertility. 

### 4.3. Supplementation of AMSCs Derivatives

Amniotic-membrane-derived MSC derivatives have also been used in canine sperm cryopreservation [172,173]. A CM derived from AMSCs (AMSC-CM) has been characterized and used at different concentrations in the freezing medium [173]. CM is obtained through stem cell starvation, during which the cells release important paracrine factors involved in cell protection [197,198,199]. Even though it contains extracellular vesicles, the use of CM itself is more advantageous as it is less expensive to obtain, and the current extracellular vesicle extraction methods do not offer a satisfactory yield [200]. Therefore, CMs have been used in many clinical trials [201]. In canine sperm cryopreservation, 10% AMSC-CM was sufficient to recover the post-thaw sperm functions, and sperm motility, membrane integrity, mitochondrial function, and viability were enhanced in the treated groups. Moreover, some of the 86 proteins present in the AMSC-CM were involved in pathways related to various processes, including cell repair, sperm motility and metabolism, and cell defense [202,203,204,205,206,207]. Therefore, MSC-CM addition to the cryopreservation extenders is safe and can produce positive post-thaw outcomes. However, as there are no in vivo and in vitro fertility assessments using MSC-CM, more studies need to be conducted to evaluate the effects of MSC-CM on fertility.

Extracellular vesicles, and more precisely exosomes, have been extracted from AMSC-CM and used in canine sperm cryopreservation [172]. However, only low concentrations, ranging from 0 to 2 µg/mL, have been tested, with no effects observed [172]. Further studies using higher concentrations, similar to those of Ad-MSC exosomes [170], combined with the characterization of AMSC-derived exosomes are warranted.

## 5. Conclusions

Numerous studies have attempted to address the deleterious effects of sperm cryopreservation, including post-thaw low motility, viability, and fertility, due to the damage caused during the freeze–thawing procedures. As oxidative stress is one of the main results of osmotic changes, various antioxidants have been used in canine sperm cryopreservation. Despite the associated risk of cross-contamination, the conventional methods for canine sperm cryopreservation have used whole egg yolk for years. To avoid the risks associated with the use of whole egg yolk, different substitutes, including egg yolk derivatives and soybean lecithin, have been investigated. However, the extraction methods used to obtain these chemicals are complex and expensive, limiting their clinical application. Recently, the effects of MSCs and their derivatives on canine sperm have been evaluated, and they have proven promising as freezing extender supplements. Given their wide therapeutic effects, MSCs or their derivatives warrant further research for application in sperm cryopreservation, with particular focus on finding ways to overcome the current limitations associated with their use and elucidate the precise underlying mechanisms of their effects. In addition, although the currently available data are promising and interesting, many further experiments, specifically in vivo assays, are imperative to confirm the actual clinical effects of these chemicals on fertility.

## Figures and Tables

**Table 1:** List of antioxidants used for sperm cryopreservation in dogs and their effects in different species.

Antioxidant	Species	Dosage	Results
Astaxanthin	Boar	2 μM	Protected post-thaw sperm motility, membrane and acrosome integrity. Inhibited lipid peroxidation. Regulation of membrane fatty acid composiion. Enhanced IVF efficiency and embryonic development [28]. Supplementation of 0.5 μM decreased apoptotic-like changes. Supplementation of 15 μM had a negative effect on sperm parameters [29].
	Canine	1 µM	Protected post-thaw sperm kinematic parameters, viability, mitochondrial activity, plasma membrane, chromatin and acrosome integrity [30].
	Miniature Pig	500 µM	Protected post-thaw sperm motility and progressive motility. Reduced ROS production [31].
	Sheep	2 µM, 4 µM	Protected post-thaw sperm viability and plasma membrane integrity. Decreased acrosome abnormalities and malondialdehyde formation [32].
Resveratrol	Boar	50 µM	Protected post-thaw sperm progressive motility, membrane and acrosome integrity, mitochondrial activity, activities of enzymatic antioxidants, and phosphorylation of AMPK [33].
	Bovine	50 μM	Protected membrane integrity and antioxidant capacity. Decreased ROS production, and capacitation-like changes. Improved in vitro fertilizing ability [34].
	Canine	200 µM	Protected post-thaw sperm motility, viability, plasma membrane, acrosome and chromatin integrity, and mitochondrial activity [35].
	Equine	5 μM	Protected post-thaw DNA and membrane integrity, total and progressive motility, and viability in subfertile stallions [36].
	Human	0.1 mM, 1.0 mM, 10.0 mM	Prevented lipid damage in a non dose-dependent manner [37].
Quercetin	Bovine	25 μg/mL	Protected post-thaw DNA integrity. No effects on post-thaw sperm kinematic parameters, and plasma membrane integrity [38].
	Canine	5 μg/mL	Protected post-thaw kinematic parameters and fertility [39].
	Equine	0.1 mM	Protected post-thaw total and progressive motility. Reduced oxidative stress [40]
	Human	50 μM	Protected post-thaw kinematic parameters, viability, and DNA integrity [41].
Myoinositol	Canine	1 mg/mL	Protected sperm motility, kinematic parameters, and membrane integrity. Reduced chromatin damage and apoptosis-like changes [42].
	Equine	30 mM	Amplitude of lateral head displacement increased [43].
	Human	2 mg/mL	Progressive motility percentage protected in normozoospermic, oligo-astheno-teratozoospermic men. Mitochondrial function improvement in patients with impaired sperm parameters. Decreased DNA fragmentation and lipid peroxidation [44,45,46].
		1 mg/mL	Increased cryosurvival rate [47].
	Sheep	5 mM, 10 mM	No significant enhancement [48].
Curcumin	Boar	0.25 mmol/L, 0.50 mmol/L	Protected post-thaw progressive motility and acrosome integrity [49].
	Bovine	0.5 mM	Protected post-thaw plasma membrane integrity and oxidative defense. Reduced percentage of abnormal sperm [50].
	Canine	2.50 mM	Protected post-thaw DNA integrity and oxidative defense [51].
	Human	20 μM	Protected post-thaw progressive motilty. Reduced DNA fragmentation and intracellular ROS [52].
Iodixanol	Bovine	2.50%	Protected post-thaw motility, progressive motility, viability, and acrosome and membrane integrity [53,54]. Protection against oxidative stress [55].
	Canine	1.50%	Protected frozen–thawed motility. Decreased capacitation, protamine deficiency, and apoptosis-like changes. Reduced mitochondrial reactive oxygen production [56].
	Equine	5%	Protected post-thaw progressive motility, plasma membrane, and DNA integrity [57].
	Rat	1%, 2%	Protected post thaw motility [58].
	Sheep	5%	Protected post-thaw progressive motility, morphology, and acrosome and membrane integrity [59,60].
Spermine	Bovine	Associated with a nitric acid donor, 10 μM	Protected frozen–thawed sperm motility, viability, membrane integrity, and decreased lipid peroxidation [61].
	Canine	5.0 mM	Protected post-thaw kinematic parameters, and membrane integrity. Decreased reactive oxygen species production, and cryocapacitation [62].
	Equine	1 mg/mL, 2 mg/mL	Decreased capacitation and DNA fragmentation index [63].
Kinetin	Canine	50 μM	Reduced sperm post-thaw oxidative damages. Protected post-thaw motility, viability, and membrane integrity [64].
	Sheep	50 μM, 100 μM	Protected cooled sperm antioxidant activity, kinematic parameters, viability, and plasma membrane. Decreased lipid peroxidation [65].
Melatonin	Bovine	1 mM, 2mM, 3 mM	Protected post-thaw motility, and antioxidant capacity. Reduced lipid peroxidation [66,67].
		0.1 mM	Protected post-thaw plasma membrane, acrosome region, and ultrastructure integrity [68].
	Canine	0.1 mM, 0.25 mM	Protected post-thaw membrane and acrosome integrity [69].
	Chicken	10^−3^, 10^−6^ M	Decreased lipid peroxidation, DNA fragmentation, and apoptosis-like changes. Protected post-thaw motility [70].
	Equine	1 μM	Higher mitochondrial membrane potentials, and protected membrane integrity [71]. Reduced lipid peroxidation [72].
	Goat	20.0 µg	Protected post-thaw motility [73].
	Human	0.01 mM, 3 mM	Protected post-thaw motility, progressive motility, and viability. Decreased intracellular reactive oxygen species, malondialdehyde, and caspase-3 activity [74,75,76].
	Mouse	0.125 mg/mL [77]	Protected post-thaw progressive motility, and anti-apoptotic gene expression [77].
	Rabbits	0.1 mM	Protected post-thaw motility, membrane and acrosome integrity, and mitochondrial membrane potential [78].
	Sheep	1 mM	Protected post-thaw motility, viability, intracellular ATP concentrations, and DNA integrity [79].
Metformin	Canine	50 µM	Protected post-thaw motility, oxidative stress defense, and quality-related markers [80].
	Mouse	5000 µM	Enhanced AMPK activity, and in vitro fertilization success [81].
Olive-derived antioxidants	Canine	10 μg/mL, 30 μg/mL, 50 μg/mL and 70 μg/mL	Protected post-thaw kinematic parameters, viability, plasma membrane integrity, and oxidative defense. Reduced DNA damage [82]
	Sheep	10 μg/mL, 30 μg/mL, 50 μg/mL and 70 μg/mL	No effects on post-thaw sperm kinematic parameters. Reduced lipid peroxidation [83].

**Table 2:** Use of mesenchymal stem cells in male reproduction and ART.

Mesenchymal Stem Cells	Form	Species	Treatment Type	Effects on Sperm
Adipose-derived MSCs	Cells	Canine	Cryopreservation	Protected post-thaw sperm motility, viability, membrane, and acrosome and chromatin integrity [171].
	Cells	Rats	Infertility	Reestablishment of spermatogenesis, and restoration of fertility [166].
	Cells	Rats	Testicular injury	Protected progressive motility and vitality. Activated Akt/GSK3 axis and stimulated glucolysis [179].
	Exosomes	Canine	Cryopreservation	Protected post-thaw sperm plasma membrane and chromatin integrity, motility, and viability [170].
Amniotic-membrane-derived MSCs	Conditioned medium	Canine	Cryopreservation	Protected post-thaw sperm plasma membrane integrity, motility, mitochondrial activity, and viability [173].
	Exosomes	Canine	Cryopreservation	No effects on post-thaw sperm quality-related parameters [172].
Bone-marrow-derived MSCs	Cells	Rats	Infertility	Restoration of fertility [180].
	Microvesicles	Rats	Cryopreservation	Protected post-thaw sperm viability, progressive motility, and antioxidant capacity. Reduced levels of necrosis, and apoptosis. Increased expression of surface adhesion molecules [174].

MSC, mesenchymal stem cells; AKT, protein kinase B; GSK3, glycogen synthase kinase 3.
